# Regulatory and policy considerations for the implementation of gene drive-modified mosquitoes to prevent malaria transmission

**DOI:** 10.1007/s11248-023-00335-z

**Published:** 2023-03-15

**Authors:** Stephanie L. James, Brinda Dass, Hector Quemada

**Affiliations:** 1 GeneConvene Global Collaborative, Foundation for the NIH, Bethesda, MD USA; 2 Kalamazoo, MI USA

**Keywords:** Gene drive, Mosquito, Regulation, Policy, Genetically modified

## Abstract

Gene drive-modified mosquitoes (GDMMs) are being developed as possible new tools to prevent transmission of malaria and other mosquito-borne diseases. To date no GDMMs have yet undergone field testing. This early stage is an opportune time for developers, supporters, and possible users to begin to consider the potential regulatory requirements for eventual implementation of these technologies in national or regional public health programs, especially as some of the practical implications of these requirements may take considerable planning, time and coordination to address. Several currently unresolved regulatory questions pertinent to the implementation of GDMMs are examined, including: how the product will be defined; what the registration/approval process will be for placing new GDMM products on the market; how the potential for transboundary movement of GDMMs can be addressed; and what role might be played by existing multinational bodies and agreements in authorization decisions. Regulation and policies applied for registration of other genetically modified organisms or other living mosquito products are assessed for relevance to the use case of GDMMs to prevent malaria in Africa. Multiple national authorities are likely to be involved in decision-making, according to existing laws in place within each country for certain product classes. Requirements under the Cartagena Protocol on Biodiversity will be considered relevant in most countries, as may existing regulatory frameworks for conventional pesticide, medical, and biocontrol products. Experience suggests that standard regulatory processes, evidence requirements, and liability laws differ from country to country. Regional mechanisms will be useful to address some of the important challenges.

## Introduction

Gene drive-modified mosquitoes (GDMMs) hold promise as new tools for control and elimination of malaria and other mosquito-borne diseases (Eckhoff et al. 2016; North et al. 2020; Metchanun et al. 2021). The efficacy of GDMMs for preventing disease transmission has not yet been demonstrated in field trials, and how best to conduct such trials remains the subject of considerable discussion. Yet it is not too early to begin considering the requirements for implementation (post-investigational use) of GDMMs as public health tools since these could have substantial ramifications for upstream research and development. As a live genetically modified product with the potential to spread and persist in the environment, GDMMs aim to achieve area-wide control with characteristics that differ substantially from current insecticide-based mosquito control methods, raising some different regulatory and policy considerations for their development and implementation.

Gene drive promotes or favors the inheritance of certain genes from generation to generation (Alphey et al. 2020) and can be used to introduce new traits rapidly through an interbreeding population. In engineered gene drive systems, the modification responsible for the new trait (the effector mechanism) might involve altering the sequence of existing genes, disabling or excising an existing gene, or introducing new genes or genetic elements into the mosquito genome. These systems aim either to reduce the size of the population of vector mosquitoes by inhibiting their reproduction or survival (population suppression) or to modify the mosquitoes to make them less competent to transmit a pathogen (population replacement) (World Health Organization 2021a). Self-sustaining drives are intended to persist, passing the modification on through subsequent generations indefinitely. Because of the properties of super-Mendelian inheritance, many self-sustaining drives are expected to spread widely within interbreeding mosquito populations. Self-limiting drive systems also have been described, which aim to impose some temporal restriction on the persistence of the modification so that it will eventually disappear from the target mosquito population. Another version under investigation, termed localizing or confined drive, intends to impose some spatial restriction on the spread of the modification through the target population (World Health Organization 2021a; Wang et al 2022).

According to the recommended development pathway (World Health Organization 2021a), widespread implementation of GDMMs as public health tools would be considered only after adequate field testing demonstrates their safety for health and the environment as well as their potential to reduce vector mosquito populations and/or prevent disease transmission. Implementation will consist of producing GDMMs in an appropriately regulated facility and systematically releasing them into the environment where they will interbreed with wild mosquitoes, thus establishing the introduced trait in the local population of the target species for some period of time. Such implementation is expected to be conducted under the authorization and oversight of national regulatory authorities and may involve authorities responsible for determining national or regional disease control priorities (World Health Organization 2021a). It is likely that there will be a period of post-implementation monitoring for safety, efficacy, and/or overall performance in the context of other malaria interventions.

Some have raised concerns that gene drive technologies pose novel risks for which current regulatory systems are unprepared (e.g., Oye et al. 2014; National Academies of Sciences, Engineering, and Medicine 2016; Meghani and Kuzma 2018; Rabitz 2019; Dolezel 2020), although others suggest that current paradigms will be adequate with some improvements (e.g., EFSA Panel on Genetically Modified Organisms et al. 2020; Romeis et al 2020; Peterson and Rolston 2022). Many of the concerns raised have broadly referenced the potential challenges for risk assessment, the need for inclusion of public input into regulatory decision-making, and the potential for movement of gene drive-modified mosquitoes across national boundaries. While issues such as risk assessment and stakeholder engagement continue to be addressed elsewhere (as reviewed in World Health Organization 2021a), here we focus on a set of practical regulatory and policy questions related to the process of new product approval/registration of gene drive products for the specific use case of GDMMs for the control and elimination of malaria in Africa, an application for which research is rapidly advancing. However, the considerations raised here also may be germane to other proposed uses of gene drive technologies in public health, agriculture and conservation.

### Defining regulatory concepts

For commercial use, the product is considered to be the entity intended to be deployed. For GDMMs, this definition would translate to the live genetically modified mosquito products which, when released into the environment, are intended to pass the modification into the local population of compatible mosquitoes at a ratio greater than that expected based on Mendelian genetics (super-Mendelian inheritance). It is useful to look to the regulation and registration of other genetically modified (GM) and/or live mosquito products for precedents that might apply to GDMMs. For GM crops, the transgenic organism traditionally has been treated as the regulated product, although in certain cases gene-edited products have been exempted^1^ (Turnbull et al 2021). It has been proposed that the GDMM product be defined as the transgenic mosquitoes (adults or any other life stage) carrying the engineered gene drive system (James et al. 2018). This is in agreement with inclusion of gene drive-modified organisms as living modified organisms (LMOs) under the Cartagena Protocol.^2^ With GDMMs, the transgenic construct is intended to spread to some extent, depending on characteristics of the gene drive system, to wild sexually compatible mosquito populations and species by interbreeding. For other live GM mosquitoes containing a heritable, albeit non-driving, construct, the product is considered to be those transgenic mosquitoes (adults or other life stage) that are produced under controlled conditions for purposeful release,^3^^,^^4^^,^^5^Consistency would dictate extension of this same definition to GDMMs (Box 1).

**Box:**
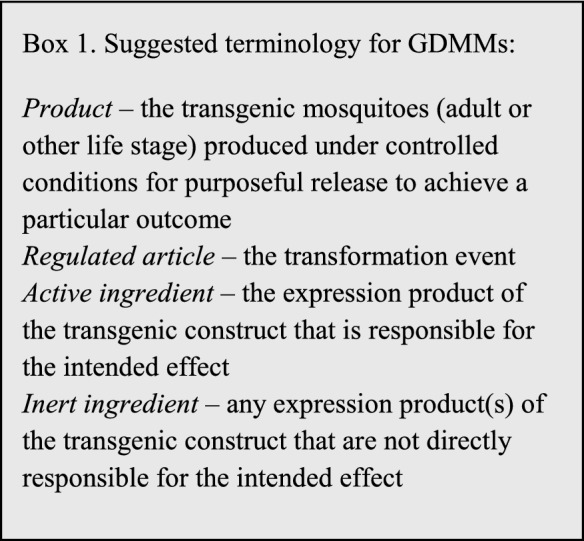
Suggested terminology for GDMMs

As for other LMOs, such as GM crops, the regulated article is expected to be the transformation event, involving stable integration of the transgenic construct leading to functional expression in the organism (OECD 2010), but regulatory approval may be requested and granted for the article and its derived products (which could include progeny in the same species or those resulting from sexual crossing into other genetic backgrounds). Thus, the same approved article may be used in more than one GDMM product if introgressed into that product.

The active ingredient for drugs has been defined by the US Food and Drug Administration (FDA) as any component responsible for the drug’s direct effect or a form that can bring about the intended effect or specified activity,^6^ whereas for pesticidal products the US Environmental Protection Agency (EPA) defines active ingredients as the chemicals (conventional, antimicrobial or biopesticidal) that act to control a pest.^7^ For live mosquito products, EPA has considered the active ingredient as the novel introduced and expressed component. For example, in a non-GM live mosquito product containing intentionally introduced *Wolbachia* bacteria for population suppression, the EPA determined the active ingredient to be the introduced *Wolbachia* in the live *Aedes albopictus* mosquito.^8^ For a different *Wolbachia*-based technology, in which the bacteria are intended to be inherited by wild progeny of the modified mosquitoes in a self-sustaining population replacement mechanism, the Australian Pesticides and Veterinary Medicines Authority determined to regulate the *Wolbachia* as a substance that modifies the physiology of the mosquito (De Barro et al. 2011).

With relevance to GM crops, the US EPA has defined a plant-incorporated protectant as the "pesticidal substances produced and used in living plants and the genetic material necessary for the plant to produce the substance”.^9^ This definition of a pesticidal product has been further extended to genetically modified mosquitoes. Thus, in a GM mosquito product for population suppression, the active ingredient was determined by the EPA to be a protein with biopesticidal properties expressed in the released live mosquito product by the transgenic construct (See footnote 4).^10^ Specifically for Oxitec's OX5034 *Aedes aegypti*, the tetracycline-repressible transactivator protein variant (tTAV-OX5034), which prevents female offspring from surviving, was recognized as the active ingredient, while the DsRed2-OX5034 marker protein was identified as an inert ingredient. In its risk assessment, EPA also considered the vector pOX5034, which is the genetic material necessary for production of these proteins in vivo.^11^ According to these precedents, the active ingredient for GDMMs would be the expression product of the transgenic construct responsible for the intended effect in the released mosquitoes. For example, this may include expressed drive components (e.g. Cas9) and/or any cargo genes that contribute to the specified effect of the GDMM as described in the product definition. Introduced genetic material necessary for production of the expressed proteins responsible for the intended effect also would be considered in the risk assessment for regulatory approval.

### Regulatory pathways for GDMM products

Most countries where GDMMs might be used for control of mosquito-borne diseases are Parties to the Cartagena Protocol on Biosafety to the Convention on Biological Diversity (CPB) (Secretariat of the Convention on Biological Diversity 2000), an international agreement concerning safe handling, transport and use of LMOs. LMOs are described in the CPB (Article 3)^12^ as any living organism (any biological entity capable of transferring or replicating genetic material…) that possesses a novel combination of genetic material obtained through the use of modern biotechnology, a definition generally considered equivalent to GM organisms (GMOs). As mentioned above, it has been concluded that GDMMs meet the LMO definition) (See footnote 2). In countries that are Parties to the CPB, GDMMs proposed as public health tools therefore are expected to enter into the regulatory pathway under biosafety oversight as described in the CPB. Article 19^12^ instructs countries to designate a competent national authority to make decisions about importation and use of LMOs. The language of Article 19 allows for more than one competent authority and responsibility for biosafety approval may reside within a single national Ministry or be shared by a group of Ministries. In many countries, the National Biosafety Authority traditionally is housed within the Ministry of Environment, Ministry of Agriculture, or Ministry of Science. However, it is recognized that more than one competent authority can be designated, depending on the nature of the LMOs and their anticipated use (Mackenzie et al 2003), which suggests the possibility of a primary role for the Ministry of Health, Ministry of Science and Technology, or other relevant national authority for GDMMs. Therefore, multiple Ministries may be involved in biosafety decision-making and countries may use the National Biosafety Authority to facilitate interactions among them.

Some countries that are not CPB signatories have adopted a product-based regulatory process that does not distinguish a separate biosafety pathway for GMOs. For example, Canada generally has a product-based regulatory system^13^ (Turnbull et al 2021). According to current guidance in the USA,^14^ GDMMs intended to reduce populations of vector mosquitoes are to be regulated as pesticides by the EPA, whereas products intended to reduce the pathogen load within mosquitoes or prevent mosquito-borne disease in humans will be regulated by the US FDA. Any GMO that could pose a plant or animal pest risk likely will be regulated by the US Department of Agriculture. These regulatory authorities are not mutually exclusive and could overlap, thus resulting in oversight by more than one agency.

### Precedents for new product approval at the national level

Assuming that successful demonstration of efficacy and safety in field trials results in a decision to incorporate GDMM products into national or regional control plans, GDMM products will need to develop plans analogous to those for commercialization of other types of products. Although the testing phases preceding GDMM implementation will have been conducted under relevant local and national regulatory oversight (discussed in World Health Organization 2021a), it is likely that this will have been accomplished under research or experimental use permits. However, national regulatory policies may involve a more expansive process for registration or final approval for placing a new product on the market for widespread and systematic use, as in the context of national disease control programs.

Current country regulatory authorities and regulations in place for GMOs are likely to be considered for GDMMs. Regulatory processes for GMOs in most countries were originally developed for GM crops (Akinbo et al. 2021; Quemada 2022). In most countries, GM crops must obtain biosafety approval from the national competent authority for GMOs, and then are subject to a national variety registration process, which applies to all new crop varieties. This registration process tests performance of the new varieties to decide whether they are suitable to be grown by farmers. The process involves a set of national performance trials, which are conducted in approved locations by national authorities located in the Ministry of Agriculture. However, a GM variety cannot enter such trials until biosafety approval is given to grow the GM crop in the presence of non-GM varieties. For GM crops, the competent National Biosafety Authority might be housed in the Ministry of Agriculture or in another Ministry. Either way, the GM crop must follow a dual pathway involving approval by both biosafety and product performance-based regulators. This existing regulatory pathway for GM crops suggests that GDMM testing and approval could also involve multiple regulatory authorities that have jurisdiction over certain product categories, regardless of their GM status. For example, a dual pathway could include the National Biosafety Authority (for biosafety approval) and the national regulatory authority with a legal mandate most relevant to oversight of the product’s final use claim.

In addition to GM crops, a few countries now have some experience regulating GM (but not gene drive-modified) insects, and efforts are underway in some other countries to adapt their biosafety regulatory processes to include oversight of GM insect products. Perhaps the regulatory experience most related to GDMMs to date has been with the self-limiting (heritable but non-driving) GM technologies developed for *Ae. aegypti* mosquitoes by Oxitec^15^ in Brazil. Brazil is a Party to the CPB, and generally has an enabling environment for new GM technologies. It presently is the only country where GM mosquitoes have been approved for commercial release. Oxitec received permission for field testing of GM mosquitoes from CTNBio (Comissão Técnica Nacional de Biossegurança, órgão do Governo Federal, based in the Ministry of Science, Technology, Innovations, and Communications), the technical entity responsible for conducting risk assessment of GMOs. CTNBio includes representatives from multiple ministries^16^ and its safety decisions are binding on those ministries.^17^ Field testing subsequently demonstrated that sustained inundative releases substantially reduced the local *Ae. aegypti* populations at trial sites (Spinner et al 2022). CTNBio has granted biosafety approval for commercial release of the second generation strain of the Oxitec Friendly™ mosquitoes^18^ as well as a similar fall armyworm product^19^ (Reavey et al. 2022). Registration and enforcement of GM products in Brazil is generally the purview of the relevant national agency(ies) dealing with health, environment, or agriculture (Velini et al. 2017; Andrade et al. 2018), with the Brazilian National Health Surveillance Agency (ANVISA) being the governmental body responsible under the Brazilian Biosafety Law of 2005 for the registration and commercial supervision of GMOs with direct implications for human health. However, the Oxitec GM mosquito products currently are being registered as pesticides with solely entomological claims and without any health claim. ANVISA does not have regulatory oversight over pesticides that are macro-organisms, such as mosquitoes. Therefore, in this specific case, CTNBio was the only regulatory agency with jurisdiction and its biosafety approval was sufficient for commercial release. CTNBio has published rules for commercial release and monitoring of GMOs in its Normative Resolution No. 32.^20^ Currently, Oxitec’s second generation GM *Ae. aegypti* mosquito product is being marketed for use by municipalities and also for direct-to-consumer sale in Brazil.^21^

In the US, the EPA has issued experimental use permits to allow testing of Oxitec’s Friendly™ mosquitoes in Florida^22^ and more recently California.^23^ The EPA also has registered the (non-GM) *Wolbachia*-based MosquitoMate population suppression product as a pesticide (See footnote 8). If a developer wishes to test the epidemiological efficacy of a GDMM product, which is a stated requirement for WHO recommendation (World Health Organization 2020a), it is likely that FDA also would play a regulatory role even for those classified as pesticides, as it does for other human and animal health products (including clinical trials).

These examples indicate that the pathway to approval of new GDMM products for widespread release may well differ among countries according to the intended use of the product and the relevant legal mandates and precedents. Developers will be expected to specify the claims for the product and intended use, which will be important in determining the regulatory pathway, and supply the relevant evidence supporting the claims. National laws in the individual country then will determine specifics of jurisdiction according to the claims, such as relevant regulations and which Ministries and Agencies have oversight. For example, if classified as a medical product, final approval for commercialization might logically fall within the Ministry of Health. The Ministry of Health can be expected already to have been heavily involved in oversight of field trials for disease efficacy for a GDMM public health or human health claim (World Health Organization 2021a), and likely also to have participated in biosafety determination. But if the GDMM product is classified as a pesticide, responsibility for final full approval might fall within the Ministries of Health, Agriculture and/or Environment depending upon national laws^24^. Other types of live mosquito products, including SIT and *Wolbachia*-based population replacement, also have experienced different regulatory pathways in different countries.

### Applicant responsibilities for new product approval

As the applicant, the GDMM developer, manufacturer or implementer will bear a number of responsibilities. First, there will be costs associated with the process of full product approval.^24^ Individual countries may charge registration fees, and there may be consultant, legal, or other costs. This would be in addition to the costs of testing requirements for a new product. As with any product, an expectation to repeat field testing of GDMMs within each country as a pre-requisite to product registration could be time-consuming and costly. Therefore, early consideration of evidence requirements and data portability, prior to field testing, will be important.

The GDMM developer or manufacturer also will be responsible for quality management of the product. Rearing of GDMMs can be compared to manufacturing of other types of public health products. It is expected that, just as for other types of new products, those seeking to bring GDMMs to market will be required to provide sufficient information to allow the regulator(s) to determine whether the product is safe and effective for its proposed use and whether the manufacturing methods are adequate to preserve the product’s integrity across release lots/batches. Methods and criteria must be established in advance for determining whether a GDMM product is acceptable for release (analogous to release specifications for other products^25^^,^ 26). Challenges can be anticipated in translating familiar requirements for medical or pesticide products (such as identity, potency, quality and purity) to GDMMs. Standards for GDMMs can be informed by practices common to other live insect products, however, such as those used in Sterile Insect Technique (SIT)^27^ for which there exist long experience and documented processes^28^ (World Health Organization and International Atomic Energy Agency 2020; Dyck et al. 2021) as well as the newer GM and *Wolbachia*-based mosquito control methods (Table 1). Information that might be required within a regulatory dossier for regulatory evaluation and decision-making on GM biocontrol products has been suggested (Tonui et al. 2022).

**Table 1:** Potential manufacturing standards for gene drive-modified mosquitoes

Standard release criteria	Description for other public health products^*,**^	Interpretation for GDMMs
IDENTITY/PURITY	Determination of which ingredients the product must contain and which it may contain	Description of the composition, copy number, and location of the transgenic construct
POTENCY/STRENGTH	Clinical efficacy, percentage of active ingredient in the product	Performance, competitiveness of transgenic mosquitoes with regard to their wild counterparts Strength of the gene drive construct, measured as spread through a population
QUALITY	Ability to demonstrate consistency in meeting identity and potency standards	Ability to demonstrate consistency in meeting identity and performance standards

*See for example https://www.ema.europa.eu/en/documents/scientific-guideline/ich-q-6-b-test-procedures-acceptance-criteria-biotechnological/biological-products-step-5_en.pdf

**See for example https://www.fda.gov/food/hazard-analysis-critical-control-point-haccp/haccp-principles-application-guidelines#execsum

It is likely that new product approval will require the applicant to propose a mechanism for monitoring and reporting of adverse events (and possibly continued product efficacy) following releases. Such post-implementation monitoring may be particularly targeted to any risks identified as non-negligible in the risk assessment. The World Health Organization (WHO) has provided recommendations for post-implementation monitoring and surveillance (World Health Organization 2021a), which could be customized to the particular GDMM product and use. The plan for adverse event reporting will need to specify what types of events will be monitored, at what frequency and duration, as well as what types of actions could be anticipated as a response to address the adverse event.

A corollary to monitoring responsibility is liability. It will be important to understand whether, and if so how, the developer, registrant, manufacturer, and/or implementer^29^ is liable for any adverse effects resulting from the GDMM product. Currently, 52 countries, including several in Africa, are Parties to the Nagoya-Kuala Lumpur Supplementary Protocol on Liability and Redress to the CPB.^30^ This protocol pertains to damage to the conservation and sustainable use of biodiversity, and requires that “response measures are taken in the event of damage resulting from living modified organisms, or where there is sufficient likelihood that damage will result if timely response measures are not taken”. According to the predicted potential for a GDMM product to move across national boundaries, liability claims may extend to neighbors of the implementing country. Countries will incorporate responsibilities for these goals into their own laws individually and may interpret requirements of these terms, including the definition of damage, differently. National liability laws will be an important determinant of receptivity to emerging technologies. For example, under fault liability those conducting the GDMM release would only be liable if they failed to conduct appropriate risk assessment to allow the government to make an informed decision about release, while the authorizing government would otherwise bear the responsibility. However, strict liability could impose costs resulting from an adverse event on those conducting the release regardless of their good faith effort to comply with risk assessment obligations (Rabitz 2019). Thus, in planning for implementation, it will be important to determine how liability laws apply and how claims, including any related to transboundary movement, might be managed. Trial and/or product liability insurance, as offered for other types of products, is a consideration.

It is important to note that, in addition to the environmental risk assessment of specific products that is a standard requirement of regulatory oversight according to the CPB and national laws, additional legal frameworks may apply in African countries. These include a Strategic Environmental Assessment, which supports political and policy level decision-making regarding a general type of intervention (for example, GDMMs), as well as an Environmental and Social Impact Assessment, which examines the broader scope of potential health, environmental and socioeconomic impacts of a specific project 31^,^ 32 (Rossouw et al. 2000; Connolly et al. 2022). Under these assessments, opportunities exist to consider potential impacts within a transboundary context.^33^

### Transboundary movement

GDMMs invoke some particularly complex questions with respect to product approval. One is the question of whether a separate product approval would be required within each country that eventually might be affected by a product that intentionally or independently crosses its national boundaries over time.

With regard to the ability of GDMMs to move autonomously across political boundaries or of the transgenic construct to move across borders via interbreeding, it is of interest to compare requirements for release of live insects for agricultural biocontrol as these also are able to disseminate autonomously. The usual procedure in that case is to submit a regulatory dossier only in the country where the release is proposed. Although it is recognized that biological control agents can cross borders, this is not normally a cause impacting the final decision of the country where the release is proposed. Indeed, transboundary movement of the control agent may be considered beneficial by a receiving country dealing with similar unwanted pests. However, responsible national authorities are encouraged to communicate details of an intended release that may affect neighboring countries.^34^

The fact that GDMMs are GMOs subject to requirements of the CPB in countries that are Parties adds an extra dimension of complexity, however. Article 7 of the Protocol imposes a requirement for advanced informed agreement regarding “the first intentional transboundary movement of living modified organisms for intentional introduction into the environment of the Party of import”.^35^ Notification under Article 17 concerning “an unintentional transboundary movement of a living modified organism that is likely to have significant adverse effects on the conservation and sustainable use of biological diversity, taking also into account risks to human health” also might be considered relevant under some circumstances.

In countries that have laws regulating GMOs, a GM product must be approved to be present in the country legally. This suggests certain possibilities according to precedent:

- Both biosafety approval and product approval are likely to be required in any country where the GDMMs are placed on the market and deliberately released.
- Expectation that GDMMs will be transported or autonomously move into another country where they have not received biosafety approval would be considered a transboundary event requiring prior notification if adverse effects on the environment might be anticipated. Even if no adverse effects are anticipated, the receiving country could take steps to remove the unapproved organism if presence of an unapproved organism would be illegal there. If the GDMMs have obtained biosafety but not  necessary product approval in the receiving country, that country still might take action to remove the GDMMs if their laws prohibit dissemination of unapproved products.
- If the transgenic construct moves via interbreeding into the local mosquito population within another country where the GDMMs have not obtained biosafety and/or product approval, then again the receiving country could take steps to remove organisms containing the construct if their presence would be considered illegal according to the language of that country's regulatory laws pertaining to LMOs, which include GDMMs. Any national decision to remove the GDMMs could invoke liability requirements for whomever is deemed responsible under the affected country’s laws.

National requirements will need to be clarified as they may pertain to product distribution, for example in the case of intentional transport across national borders from a regional production facility to release sites in a neighboring country. Any particular requirements or restrictions on international transport of GDMMs should be explored before decisions about production facility location(s) are reached. Gene drive modifications may impose particular permitting requirements for shipping (Niassy et al 2022), not only due to notification requirements for transboundary movement of GMOs imposed by the CPB but also to provide the level of containment necessary to avoid inadvertent release in transit that might result in unauthorized establishment of the mosquito (American Committee of Medical Entomology 2022).

### Multilateral agreements

Given the inherent potential for transboundary movement, considerations for implementation of GDMMs extend beyond a traditional bilateral “exporter-importer” relationship (Rabitz 2021) to include the possibility of multiple receiving countries. Planning for the use of GDMMs therefore would be greatly facilitated by mechanisms to strengthen decision-making at the regional level. Article 14 of the CPB (See footnote 12) acknowledges that “Parties may enter into bilateral, regional and multilateral agreements and arrangements regarding intentional transboundary movements of living modified organisms…” and “the provisions of this Protocol shall not affect intentional transboundary movements that take place pursuant to such agreements and arrangements…” This highlights how the embrace of regional GDMM authorization and implementation programs by national governments could streamline regulatory requirements and unify transport, release, and subsequent monitoring and reporting processes.

Multilateral agreements are common and can take many forms.^36^ For addressing transboundary movement of GDMMs in Africa, the most straightforward approach could be to build upon an existing regional multilateral cooperation agreement, such as the charter establishing the Organization of African Unity/African Union,^37^ the treaty establishing the African Economic Community,^38^ or those creating the various regional economic communities^39^ and other multinational cooperative relationships.^40^ In this regard, the African Union Development Agency-New Partnership for Africa’s Development (AUDA-NEPAD) is working to establish harmonized regulatory guidelines for integrated vector management across the African continent, and these guidelines will apply to GDMMs.

Adoption of integrated mosquito management approaches has been hindered for multiple reasons, including the need for increased political backing and for better collaboration both within the health sector and with other sectors (Beier et al 2008). This has led to an increased call for intra- and intersectoral collaboration where environmental management and health education are both linked to proactive strategies for controlling new and emerging threats. In this context, need has been recognized for regulatory capacity strengthening, as well as appropriate stakeholder engagement at all levels, in preparation for decision-making on testing and implementation of all innovative vector control tools including GDMMs (e.g., Beier et al 2008; World Health Organization 2017; Glover et al 2018). To that end, and in accordance with the recommendations of the African Union High Level Panel on Emerging Technologies,^41^ AUDA-NEPAD initiated its Integrated Vector Management program as a continent-wide flagship program to ensure adequate capacity building for the African Union’s 55 member states (See footnote^42^). The program strives to ensure that there is a balance between safety of the environment and improvement of human health so that regulations are not unnecessarily so restrictive as to lose the potential health benefits of the various vector control approaches. Among other activities, the program aims to bring African countries together to decide on a uniform approach to regulating GDMMs across the continent, which could standardize the evidence needed for biosafety and product approval and foster collaboration among countries in decision-making. As currently envisioned, the program would not preclude the need for individual national product approval, although standardization of requirements could greatly simplify the process.

The first phase of the program, from 2016 to 2018, focused on raising awareness about GDMMs and other GM mosquitoes among decision makers. A series of stakeholder consultations were conducted across the five economic regions in the continent to discuss the potential benefits and risks of these technologies to prevent malaria transmission (Teem et al 2019). Following these regional consultations, AUDA-NEPAD, with the support of the West Africa Health Organization under the Economic Community of West Africa States (ECOWAS) established the West Africa Integrated Vector Management (WAIVM) platform with the aim to operationalize a regional platform that will enable a strong collaboration among the health sector and other sectors (environment, scientific research and higher education, agriculture, trade, etc.) to effectively control disease vectors. WAIVM organized Technical Working Groups (TWGs) responsible for developing guidelines and other common technical documents that would facilitate harmonized assessment and decision-making for GDMM research. The African Biosafety Network of Expertise, a program of AUDA-NEPAD, provides services to support improvement of biosafety regulatory systems within individual countries.^43^

It is envisioned that evidence gained from the WAIVM regional platform can serve as a model for a scale-up to the continent level (Africa-IVM). Through this platform, it is expected that AUDA-NEPAD, the African Union Commission, and the African Union Centres for Disease Control and Prevention (Africa CDC) will collaborate with partners to strengthen the capacity of regulators and relevant stakeholders in Member States and Regional Economic Communities to ensure that countries and regions are able to explore the potential of new vector control approaches including GDMMs. Activities will include (i) establishment and operationalization of an Africa Vector Management Advisory Panel; (ii) establishment and operationalization of TWGs, (iii) establishment and operationalization of an Africa-IVM discussion forum, (iv) development and adoption of technical documents, and (v) guidance on international negotiations pertaining to vector control.

### Malaria intervention frameworks

It is generally assumed that implementation of GDMMs to combat malaria will be conducted by or under the oversight of a national public health program aimed at disease control and elimination, in the context of other mosquito and malaria intervention efforts (World Health Organization 2021a). However, opportunities for some form of regional oversight could be helpful for designing and managing a comprehensive GDMM implementation program, as well as addressing issues that may  create challenges for regulatory management at a strictly national level, such as transboundary movement of a single GDMM product or potential for introduction of more than one GDMM product within the same or nearby regions (James et al. 2018). Such multinational programs have proven beneficial for agricultural biocontrol programs,^44^ as well as SIT against agricultural (Enkerlin et al 2017) and public health pests.^45^ There is precedent for regional programs focused on malaria intervention (Lover et al 2017), including in Africa 46^,^ 47.

As a potential public health intervention for control and elimination of malaria, recommendations on GDMMs come under the purview of the World Health Organization’s Global Malaria Programme,^48^ which supports Member States to achieve their targets under the Global Technical Strategy for Malaria (World Health Organization 2021b). Citing an urgent need for development of new tools for control of vector-borne diseases such as malaria, the WHO issued a position statement in 2020 supporting the investigation of gene drive and other genetically modified mosquito approaches^49^ and, as mentioned above, has published guidance for all aspects of their testing (World Health Organization 2021a).

The WHO is often looked to by developing countries as a source of technical standards and has established systematic processes for drugs, diagnostics, vaccines and conventional vector control products. WHO evaluates vector control products through two complementary pathways—the Prequalification pathway and the New Intervention pathway.^50^ The prequalification process is used with products in a class for which a WHO policy recommendation exists. This pathway assesses quality, safety and efficacy of the product, as well as determining the capacity of a manufacturer to produce products of consistent quality in accordance with international standards. The WHO list of prequalified products is used by international procurement agencies, such as the Global Fund to fight AIDS, TB and malaria, to guide their purchases, and thus has been very influential for product uptake in developing countries. The WHO Vector Control Product Assessment Team (PQT/VCP)^51^ assesses vector control products and public health pesticide active ingredients to determine that they can be used safely and effectively, and consistently are manufactured to a high-quality standard. This is done by assessing product dossiers,^52^ inspecting manufacturing sites^53^ and supporting quality-control testing of products. Thus far, these activities have largely been directed toward chemical pesticides and pesticide-based products. WHO and FAO, both UN agencies, develop specifications for pesticides jointly.^54^ The specifications encompass the physical appearance of the material, its content of active ingredient(s) and any relevant impurities, physical and chemical properties, and stability in storage. Whether or how the WHO prequalification process will apply to live mosquito products remains to be clarified.

The New Intervention pathway, which complements the prequalification pathway, is intended to support deliberations on a WHO recommendation for innovative vector control products (i.e. a product type without prior WHO recommendation) (World Health Organization 2020a). Thus, the New Intervention pathway currently is most appropriate for GDMMs as a new product class. Presently, new vector control interventions are subject to evaluation by the Vector Control Advisory Group (VCAG),^55^ which focuses on assessment of the public health value of the new product. As currently stated, once data from at least two trials demonstrating epidemiological impact have been assessed by VCAG, WHO may commission a systematic review and convene a Guidelines Development Group that will consider broader issues including not only the quality of the epidemiological evidence, but also the balance of benefits and harms, resource implications, the priority of the problem, equity and human rights, acceptability and feasibility of the new intervention.^56^ Under this pathway, quality management issues are still addressed through the prequalification process. Recently proposed considerations for obtaining a permit to release GM biocontrol products may help guide applicants toward relevant issues (Tonui et al 2022).

It should be noted that WHO prequalification is not required when countries are not seeking funding from sources such as the Global Fund^57^ to support use of the product in vector control efforts. However, if countries seek the reassurance of WHO quality review of a GDMM product before making a decision to implement, it will be necessary to identify a mechanism appropriate to the characteristics of GDMMs. Clarification of WHO mechanisms for live mosquito products is currently in process, initially focused on review of *Wolbachia*-mediated population replacement products.^58^

## Discussion

Previous efforts to delineate a development pathway for GDMMs have dealt largely with requirements for research and testing and have not delved deeply into planning for implementation in the context of national or regional public health programs (James et al 2018). Yet, because GDMMs will be GMOs expected to persist and spread to some extent in the environment and intended for use as public health tools, substantial early thought must go into planning for the transition of GDMMs from testing to implementation. This analysis examines several aspects of the last step of the national regulatory approval process, registration or new product approval, which standardly sets requirements that must be met for commercial use. While it still remains to be seen how GDMMs eventually might be marketed and delivered, it is important to understand how existing regulations and policies developed for other product types may apply and to begin planning for how any challenges arising from GDMM characteristics might be addressed.

Countries that have developed regulatory pathways for other GMOs will likely be challenged with how to fit newer technologies into existing regulations. However, the relevance of both biosafety and health mandates, as well as inexperience with the features of a live insect product, may cause confusion and complicate even established regulatory processes. The more dissimilar a new GM product is from prior regulatory experience (generally GM crops) in each country, the more time it is likely to take to develop appropriate capacity and mechanisms for its review. Lack of clarity about the roles of different national regulatory agencies and uncertainty about data requirements for the regulatory submission could substantially delay regulatory evaluation and decision-making. There is a need to identify the roles and requirements of the agencies that are relevant to registration of GDMMs, especially in countries likely to be early adopters of the technology. Appropriate regulatory frameworks may need to be adapted or even created in some cases, and clear mechanisms for inter-agency cooperation may need to be established. Countries that generally are enabling to new GM technologies may be more proactive about streamlining their processes, timelines, and requirements. While it is possible that countries that have no pre-existing GMO regulations could be at some advantage as they can develop fit-for-purpose regulations and institutions, the lack of GMO regulations also may reflect a low level of overall regulatory capacity, in which case building necessary capacity can be expected to take considerable time, resources, and political will. Those developing new GDMM products would be well advised to put contingency plans in place to respond to unanticipated delays and related expenses. Early in creation of their business models, developers should undertake an extensive scoping of all the requirements at the national and local level relevant to GDMMs in locations where they wish to deliver their products, including laws, policies, guidelines, and any useful case history or precedents. This will inform the procedures to be followed, the types of evidence they must collect during prior testing phases, as well as the agencies/ministries with whom they need to initiate ongoing contacts. Proactive communication between developers and regulatory agencies can help to build understanding and allow time for clarification of national review and decision-making processes.

The possibility of transboundary movement of transgenic mosquitoes or their genes additionally suggests a need for substantial planning at the regional level. Requirements for GMOs under the CPB will be considered relevant in most countries, but exactly how these requirements apply might take into account the expectations for the specific gene drive-modified organism under consideration, which may differ according to the type of drive system (self-sustaining, self-limiting or localized). Most types of GDMMs are intended to spread to some extent and therefore might eventually cross national borders, although those carrying localizing drives are expected to be more confined and those with self-limiting drives may not persist. It is expected that GDMMs will undergo extensive risk and impact assessments in countries of initial release for evaluation of potential adverse effects. However, experience suggests each country will carry out its own approval and that standard regulatory processes and evidence requirements are likely to differ from country to country. A need for submission of dossiers to all countries where GDMM transgenes might ultimately spread and requirement for full product approval in each of these countries prior to initial release in a first country would not be in line with the history of GM crops. However, it might be possible to stage national applications according to anticipated timing of transboundary movement as judged by distance from the release site, for example based on modeling predictions of GDMM spread. Regional harmonization of data and risk assessment requirements and mechanisms for data portability and data sharing among countries could substantially simplify the processes for authorizing implementation at the national or multinational level. Initiatives such as the Africa-IVM Programme led by AUDA-NEPAD will be central to this effort.

Given the history of malaria mosquitoes developing resistance to conventional control tools, it is worthwhile to consider that the more rapid the area-wide roll-out of GDMMs the less likely it may be that resistance mechanisms become prevalent before the first GDMM products are able to fully exert their protective effects. If so, this would argue that a more harmonized and systematic multi-country approach to implementation also would provide the greatest opportunity to achieve the widely supported goals of eliminating and eradicating malaria (Feachem et al. 2019) thereby enhancing the public health benefit of GDMMs.

Even though releases of the first GDMM products are not expected for several years, planning for regional cooperation should start now. Lessons from the experience of the Innovation to Impact (I2I) program, which is working toward a collaborative registration procedure for conventional pesticide-based vector control tools to facilitate market access,^59^ suggest that such harmonization of regulatory processes will not be straightforward. Likewise, the West Africa regional biosafety program conducted jointly by the West Africa Economic and Monetary Union and ECOWAS has been working for more than ten years to develop a harmonized biosafety framework. This process was only concluded recently with the formal adoption by the Conference of the Heads of State of the ECOWAS Members states, and is yet to be operationalized. Fortunately, however, the experience from the Sahelian Committee for Pesticides, which is the regional body responsible for implementing the Common Regulations to the Permanent Inter-State Committee for Drought Control in the Sahel (CILSS) member states for pesticide homologation, sets a good example of successful regulatory harmonization. This Committee has been operational since 1994, and evaluates dossiers submitted by pesticide companies for approval for sale of chemical pesticides in the seven member states of the CILSS.^60^

If health claims are made and GDMMs are regulated as medical products, the African Medicines Agency, which serves as an AU-wide platform for coordinating and strengthening harmonization efforts for medicines regulation, could be beneficial for multinational coordination of regulatory requirements,^61^ although this effort also faces challenges (Ncube et al 2021). Other pan-African authorities, such as the Africa CDC,^62^ also could facilitate a continent-wide harmonized decision-making process on GDMMs as a public health product. Although it may not be required when countries are not seeking funding from sources such as the Global Fund to support the use of a GDMM product in their vector control efforts, a WHO recommendation may provide additional useful input for national and regional decision-making, manufacturing, and implementation planning.

While prior regulatory experience with other GMOs as well as live conventional biocontrol agents that spread in the environment affords valuable precedents for GDMMs (Romeis et al 2020), the presence of both of these characteristics within a single product undoubtedly results in a new combination of challenges. This analysis touches on some of the overarching regulatory issues that must be addressed if GDMMs are to achieve their projected potential for improving public health, and highlights the benefit of a regional approach. Many details of such an approach remain to be clarified, including how decisions will be made and who will be involved in decision-making (e.g. National Academies of Sciences, Engineering, and Medicine. 2016; Kofler et al 2018; Kelsey et al 2020; World Health Organization 2020b), as well as specific operational guidance for production, release and post-release monitoring. Early and systematic analysis of all the activities necessary to implement a particular GDMM product, including anticipated regulatory and policy considerations as well as operational requirements, can inform the development pathway and help to optimize the efficiency, effectiveness, and safety of these promising new tools for control and elimination of malaria and other mosquito-borne diseases.

## Footnotes

1 See for example https://www3.epa.gov/pesticides/chem_search/ppls/067979-00038-20210121.pdf.

2 https://www.cbd.int/doc/c/aa10/9160/6c3fcedf265dbee686715016/synbio-ahteg-2017-01-03-en.pdf.

3 ,https://www.oxitec.com/en/news/oxitecs-friendly-mosquito-technology-receives-us-epa-approval-for-pilot-projects-in-us.

4 https://www.govinfo.gov/content/pkg/FR-2020-06-09/pdf/2020-12372.pdf.

5 https://www.oxitec.com/en/news/just-add-water-oxitecs-new-friendly-mosquito-mini-capsule-technology-rapidly-suppresses-95-of-disease-spreading-aedes-aegypti-in-brazil-trial.

6 https://www.accessdata.fda.gov/scripts/cdrh/cfdocs/cfcfr/CFRSearch.cfm?fr=210.3.

7 https://www.epa.gov/ingredients-used-pesticide-products/basic-information-about-pesticide-ingredients.

8 https://www3.epa.gov/pesticides/chem_search/ppls/089668-00004-20171103.pdf.

9 https://www.epa.gov/regulation-biotechnology-under-tsca-and-fifra/overview-plant-incorporated-protectants#Overview.

10 https://www.regulations.gov/document/EPA-HQ-OPP-2019-0274-0002.

11 https://www.regulations.gov/document/EPA-HQ-OPP-2019-0274-0465.

12 https://bch.cbd.int/protocol/text/.

13 https://inspection.canada.ca/plant-varieties/plants-with-novel-traits/eng/1300137887237/1300137939635.

14 https://www.fda.gov/regulatory-information/search-fda-guidance-documents/cvm-gfi-236-clarification-fda-and-epa-jurisdiction-over-mosquito-related-products.

15 Oxitec’s second generation Friendly™ platform technology employs a gene that prevents female offspring from surviving, allowing for male-only production. This was originally developed in *Aedes aegypti* mosquitoes as a method to prevent arbovirus transmission (OX5034) but has been extended to other pest species. GM males are repeatedly released into the field in large numbers, where they find and mate with wild females of the same species. Reduction of the target population is achieved because female offspring from these encounters do not survive. Male progeny survive, carrying a copy of the self-limiting gene; in turn, these males are able to pass on the self-limiting gene to half of their offspring, of which female carriers of the gene cannot survive. The self-limiting gene therefore declines in frequency over several generations (https://www.oxitec.com/en/our-technology). The 1^st^ generation of this technology (OX513A), which resulted in nonviable male and female progeny upon mating with wild-type females and whose effect is limited to a single generation, was previously tested in Brazil (Carvalho et al. 2015).

16 http://ctnbio.mctic.gov.br/en/a-ctnbio.

17 http://ctnbio.mctic.gov.br/en/leis/-/asset_publisher/NT53w3Yb7zpx/content/lei-n-11-105-de-24-03-2005.

18 https://www.in.gov.br/web/dou/-/extrato-de-parecer-tecnico-n-6.946/2020-2582.

19 https://www.in.gov.br/web/dou/-/extrato-de-parecer-tecnico-n-7.350/2021-307396425.

20 https://www.in.gov.br/en/web/dou/-/resolucao-normativa-n-32-de-15-de-junho-de-2021-326241632.

21 https://www.aedesdobem.com.br/.

22 https://www.epa.gov/pesticides/epa-approves-experimental-use-permit-test-innovative-biopesticide-tool-better-protect.

23 https://www.epa.gov/pesticides/following-review-available-data-and-public-comments-epa-expands-and-extends-testing.

24 See for example https://innovationtoimpact.org/wp-content/uploads/2019/09/Selected-Country-Registration-Processes-for-Vector-Control-Tools.pdf.

25 See for example https://www.ema.europa.eu/en/documents/scientific-guideline/ich-q-6-b-test-procedures-acceptance-criteria-biotechnological/biological-products-step-5_en.pdf.

26 See for example https://www.fda.gov/food/hazard-analysis-critical-control-point-haccp/haccp-principles-application-guidelines#execsum.

27 SIT involves ongoing inundative releases of male insects sterilized by radiation, has been used extensively against agricultural pests, and is now being adapted to mosquitoes (https://www.iaea.org/topics/sterile-insect-technique; Oliva et al. 2021).

28 https://www.iaea.org/topics/insect-pest-control/manuals-and-protocols.

29 The same entity may play more than one of these roles.

30 https://bch.cbd.int/protocol/supplementary/.

31 https://www.cbd.int/decision/cop/?id=11042.

32 https://www.ifc.org/wps/wcm/connect/9608497e-56e8-4074-bab6-45c61a36a4ad/ESIA.pdf?MOD=AJPERES&CVID=jkCYZ3G.

33 https://unece.org/environment-policyenvironmental-assessment/text-convention.

34 https://www.fao.org/3/j5365e/j5365e.pdf.

35 https://bch.cbd.int/help/topics/en/The_Advance_Informed_Agreement_procedure.html.

36 Reviewed in https://oneill.law.georgetown.edu/wp-content/uploads/2022/07/ONeill-FNIH-Sovereignty-Implications-of-a-Pandemic-Instrument-1.pdf.

37 https://au.int/en/overview.

38 https://au.int/sites/default/files/treaties/37636-treaty-0016_-_treaty_establishing_the_african_economic_community_e.pdf.

39 https://au.int/en/organs/recs.

40 See for example https://www.mea.gov.in/Portal/ForeignRelation/east-african-community-april-2011.pdf.

41 https://www.nepad.org/microsite/african-union-high-level-panel-emerging-technologies-apet.

42 https://www.nepad.org/microsite/integrated-vector-management-ivm.

43 https://www.nepad.org/microsite/african-biosafety-network-of-expertise-abne.

44 https://www.cabi.org/about-cabi/how-we-are-managed/.

45 https://www.iaea.org/sites/default/files/publications/magazines/bulletin/bull44-1/44105561116.pdf.

46 https://endmalaria.org/sites/default/files/RBM%20SaME%20factsheet%20ENG.pdf

47 https://malariaelimination8.org/.

48 https://www.who.int/teams/global-malaria-programme#:~:text=The%20WHO%20Global%20Malaria%20Programme,2015%20and%20updated%20in%202021.

49 https://apps.who.int/iris/bitstream/handle/10665/336031/9789240013155-eng.pdf.

50 https://www.youtube.com/watch?v=k94kiQcfv8s.

51 https://extranet.who.int/pqweb/vector-control-products/what-we-do.

52 https://extranet.who.int/pqweb/assessment.

53 https://extranet.who.int/pqweb/inspection-services.

54 https://extranet.who.int/pqweb/vector-control-products/who-specifications-pesticides.

55 https://www.who.int/groups/vector-control-advisory-group/about.

56 https://apps.who.int/iris/bitstream/handle/10665/145714/9789241548960_eng.pdf?sequence=1&isAllowed=y.

57 https://www.theglobalfund.org/en/.

58 https://www.who.int/news-room/articles-detail/call-for-public-consultation-development-target-product-profiles-wolbachia-infected-aedes-aegypti-population-replacement-intervention.

59 https://innovationtoimpact.org/workstreams/country-registration/.

60 http://csp.dev4u.it/index.cfm.

61 https://au.int/en/pressreleases/20200205/african-medicine-agency-ama-treaty.

62 https://africacdc.org/.
